# Supplementary material for: Impact of ecotourism on abundance, diversity and activity patterns of medium-large terrestrial mammals at Brownsberg Nature Park, Suriname
Source: PLoS One. 2021 Jun 2;16(6):e0250390. doi: 10.1371/journal.pone.0250390 (PMC8171955; doi:10.1371/journal.pone.0250390)
Supplement: Attachment — Submitted filename: Response to Reviewers.docx [file pone.0250390.s001.docx]

**Response to Reviewers** - Impact of ecotourism on abundance, diversity and activity pattern of medium-large terrestrial mammals at Brownsberg Nature Park, Suriname

**Comments by academic editor:**

1. Please ensure that your manuscript meets PLOS ONE's style requirements, including those for file naming.

Manuscript follows PLOS ONE style.

2. Please review your reference list to ensure that it is complete and correct.

Reference list has been reviewed and the following changes have been made: volume/issue/page additions, journal correction, author corrections, formatting corrections. We added seven references to support our revised manuscript [54-56,76,81-83]. We reviewed all the references at their journal pages and against the retraction watch database (retractiondatabase.org) and found no retracted papers in our list.

3. Regarding the use of copy righted materials in Fig 1 and Fig 5:

We changed the map of South America for one from the CIA The World Factbook 2021. As stated by the CIA "The *Factbook* is in the public domain. Accordingly, it may be copied freely without permission of the Central Intelligence Agency (CIA)".

*The World Factbook 2021*. Washington, DC: Central Intelligence Agency, 2021. Political South America.
[**https://www.cia.gov/the-world-factbook/**](https://www.cia.gov/the-world-factbook/)

The maps of Suriname and the study area were created in Qgis with shapefiles that we own. Roads and trails were mapped by ourselves.

**Comments by reviewer #1:**

2 – patterns

Added “s”.

35 – should include scientific names at first mention

Included.

108 – are there more recent numbers for tourists?

Unfortunately, STINASU was unable to provide more recent numbers.

122-123 – might want to make it clear that you are referring to a diversity index given that species richness was not negatively related to tourist numbers

Sentence changed.

145-148 – it would useful to know the distribution of rainfall amounts between wet and dry seasons

Data not available for Brownsberg. However, some variation in the dryness of the dry season is reported by De Dijn et al. (2007) and is added to the text.

178 – Figure 1 – the notations on the map are hard to read; also the road to the north is not clear; in contrast, figure 5 clearly shows the roads, the lodging area, and trails; Fig 1 might be clearer if the background was not solid gray and if roads and trails were color coded as in Fig 5

Fig 1 has been fully revised.

183 – lodging facilities are not apparent to me

Tourist facilities indicated in Fig 1 now.

201 – what other cameras were used?

Reconyx PC600 cameras added to the text.

280 – remarkably high number of triggers, particularly for the three felids – any explanations for such high numbers? Also, with dual cameras, did you identify individuals for the jaguar and ocelot? Were the triggers mostly from the same individuals walking back and forth along the road?

Cameras were positioned along roads and trails, consequently producing a high capture rate of the three cats (that prefer using roads to move around). We identified the jaguars to individuals to investigate population parameters. This will be reported in another paper. In the discussion of this manuscript, we mention the density, which is quite high in most periods. Indeed, the resident jaguars triggered the same cameras often.

299 – Figures 2 and 3; if cameras in Figure 1 were numbered then the points in these figures could also be numbered, that would allow readers to see which cameras the dots refer to

Data points in Fig 2 and 3 labeled with locations.

305 – make clear that the correlation is between the RAI and number of tourists;

It is not the correlation between RAI and number of tourists. It is the correlation between the numbers of a certain mammal species per camera and the number of tourists per camera.

Added a few words in the text.

354 – I may have missed it but what are the groups for ANOSIM

Groups are in the legend at the right of Fig 6.

358 – Figure 6 – the labels are often overlapping and hard to read

Fig 6 has been revised

361-366 – seems more like discussion than results

We think that it is a good introduction/transition to the activity pattern section of the results.

403 -404 – but important to note that there was only one other site in Amazonia (Manaus) and none in the more diverse Neotropical areas closer to the Andes

This is what is published. Are there publications with a higher number of medium-large mammal species from Amazonia or closer to the Andes? If so, we will include these.

444 – actually the number of stations along trails (10) was not much less than in this study (16) and at TBS there also were 32 additional stations on two study plots

We agree that the total effort is probably sufficient to find an effect. We have thus modified our discussion point to the limited timeframe out of a year and added some literature in support of the argument.

504 – jaguars are more diurnal, particularly males, in eastern Ecuador

Text added

527-529 – but the high rates for large cats suggests lack of an effect

We showed an impact on tapir and collared peccaries too. These are important "ecosystem engineers" and their avoidance of areas with many tourists is likely to have an impact on plant composition. The big cats are still present in the area with most disturbance, however have shifted their activity mostly to the night. This is likely to have an effect on both diurnal and nocturnal prey species. We added a part on tapir and peccaries in the discussion.
